# Rheological dataset of calcium-alginate hydrogels after ultraviolet, ethylene oxide, steam, gamma, and high hydrostatic pressure treatments

**DOI:** 10.1016/j.dib.2026.113336

**Published:** 2026-10-13

**Authors:** Agnese Brangule, Paula Kaufelde, Anete Vircava, Konstantīns Logviss

**Affiliations:** a Department of Pharmacology and Pharmacotherapy, Riga Stradiņš University, Riga, LV-1007, Latvia; b Baltic Biomaterials Centre of Excellence, Headquarters at Riga Technical University, Riga, LV-1658, Latvia; c Laboratory of Finished Dosage Forms, Faculty of Pharmacy, Riga Stradiņš University, Riga, LV-1007, Latvia

**Keywords:** Soft biomaterials, Rheology, Viscoelasticity, Storage modulus, Loss factor, Linear viscoelastic region, Oscillatory rheometry

## Abstract

Sterilization-related treatments are commonly applied to hydrogel-based biomaterials, and rheological data provide a basis for describing their post-treatment material profiles. This data article presents raw oscillatory rheometry data from untreated calcium-alginate hydrogel samples and from samples exposed to ultraviolet irradiation, ethylene oxide treatment, steam sterilization, gamma irradiation, and high hydrostatic pressure treatment. The dataset was generated by amplitude sweep measurements using an Anton Paar MCR 102e rheometer with parallel-plate geometry at 37 °C. The deposited CSV files include point-by-point values for shear strain, shear stress, storage modulus, loss modulus, loss factor, torque, and complex viscosity. Corresponding plots show storage and loss moduli versus shear strain, storage and loss moduli versus shear stress, and complex viscosity versus shear strain. The files are organized by treatment group; gamma-treated samples are arranged by dose, and high hydrostatic pressure-treated samples by pressure level and number of cycles. The dataset can be reused to extract viscoelastic parameters, define the linear viscoelastic region using user-selected criteria, generate rheological profiles for untreated and treatment-exposed hydrogels, and support exploratory analysis and testing of rheological data-processing workflows.

## Graphical abstract

**Figure:**
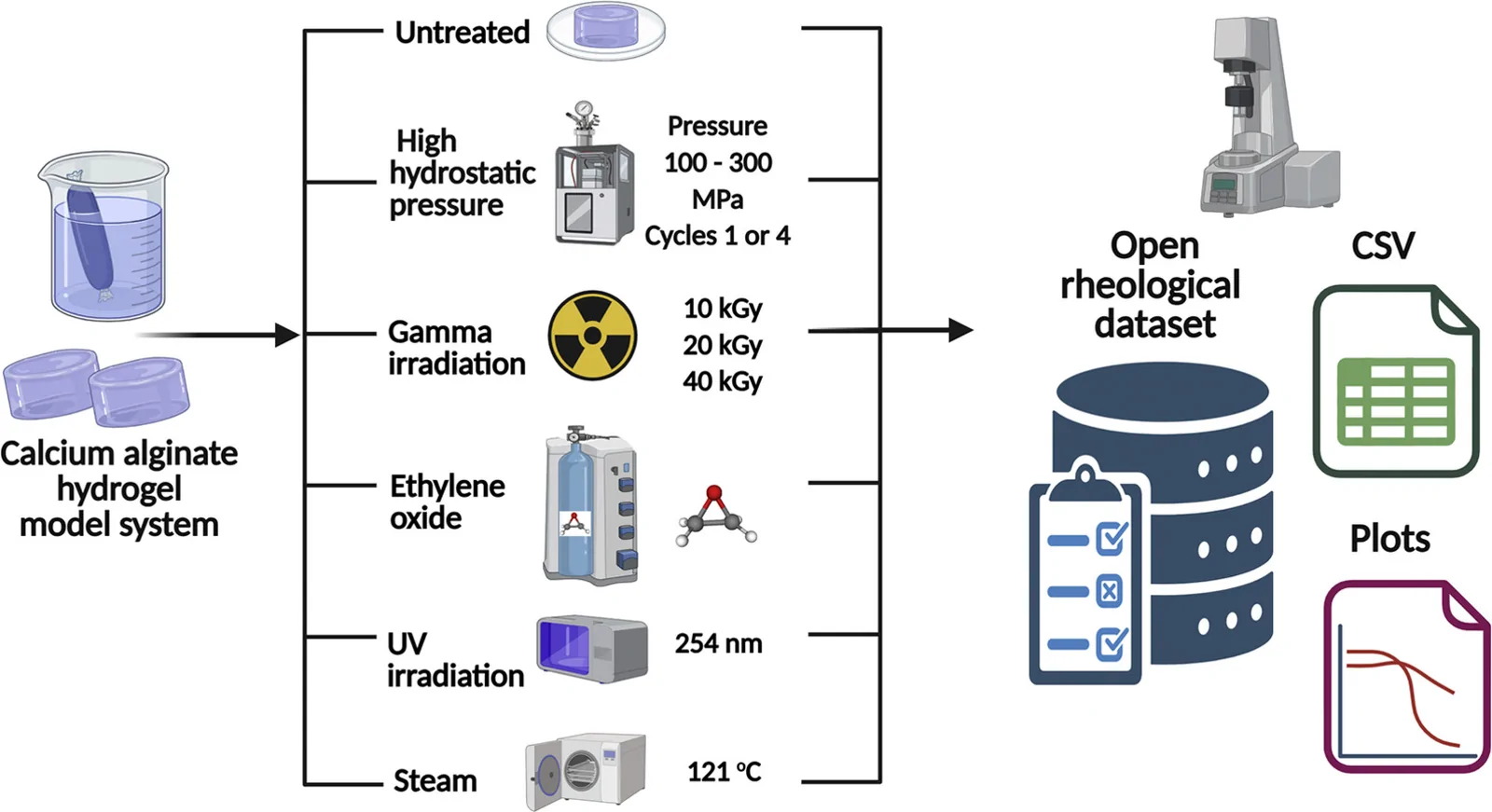

Specifications Table

**Table:** 

Subject	Engineering & Materials science
Specific subject area	Rheology and viscoelastic properties of untreated and treatment-exposed soft polymer networks
Type of data	Raw Tables (CSV files), graphs (PDF files)
Data collection	Rheological data were collected from calcium-alginate hydrogels prepared by crosslinking 2% sodium alginate with 1.5% calcium chloride and then cut into sections with a nominal thickness of 1 cm before treatment. For each treatment condition, one section from each of three independently prepared hydrogel batches was measured. Measurements were obtained for untreated reference samples and samples exposed to five treatment approaches: ultraviolet irradiation, ethylene oxide treatment, steam sterilization, gamma irradiation, and high hydrostatic pressure treatment. Rheological data were acquired at 37 °C using an Anton Paar MCR 102e rheometer with smooth 50 mm parallel plates and Julabo Corio CP-200F temperature control. Amplitude sweep tests were conducted from low to high strain at a constant angular frequency (ω) of 10 rad s^−1^ in the shear strain (γ) range of 0.01–100%
Data source location	Country: Latvia Affiliation: Riga Stradiņš University, Faculty of Pharmacy, LV-1007, Riga, Latvia
Data accessibility	Repository name: Zenodo Data identification number: DOI 10.5281/zenodo.19230556 Direct URL to data: https://zenodo.org/records/19230556
Related research article	Kaufelde, P.; Vircava, A.; Skadiņš, I.; Rubeze, K.; Proskurins, J.; Logviss, K.; Brangule, A. The Trade-Off Between Sterility and Structural Integrity in Sterilized Alginate Hydrogels. *Gels* 2026, *12*, 478. 10.3390/gels12060478

## 1 Value of the Data

• **Direct reuse for rheological parameter extraction.** The dataset provides raw amplitude-sweep measurements in CSV format of calcium-alginate hydrogels before and after ultraviolet, ethylene oxide, steam, gamma, and high-hydrostatic-pressure treatments. The files can be used to extract storage modulus, loss modulus, loss factor, complex viscosity, and the linear viscoelastic region in different data analysis environments.
• **Cross-treatment comparison of hydrogel rheology.** The data allow direct comparison of hydrogel rheological behavior after heat-, radiation-, chemical-, surface-, and pressure-based sterilization treatments. This is useful for researchers selecting processing conditions for calcium-alginate hydrogels under comparable formulation and measurement conditions.
• **User-defined processing of raw rheometry data.** With point-by-point measurement values provided, users can apply their own criteria for defining the linear viscoelastic region. This enables recalculation of plateau moduli, loss factor, and related viscoelastic parameters according to different laboratory protocols or software workflows.
• **Input for modeling and data-driven analysis.** The dataset can be used to explore relationships between treatment conditions and hydrogel rheological variables. It is suitable for exploratory regression modeling, clustering of rheological profiles, method testing, and proof-of-concept data analysis, or comparison with newly generated datasets from other hydrogel formulations. With 13 conditions and three independent material-preparation replicates per condition, the dataset is best suited for exploratory analysis, method testing, and proof-of-concept modeling rather than broadly generalizable predictive modeling.
• **Support for sterilization method selection in soft biomaterials**. The dataset can be used as the rheological component of early-stage screening of sterilization-related treatments for water-rich biomaterials, alongside microbiological efficacy and safety data.

## 2 Background

In a related research article, ultraviolet, ethylene oxide, steam, gamma, and high-hydrostatic-pressure treatments were applied to calcium-alginate hydrogels to evaluate sterility and structural integrity in the same model system [1]. Rheological characterization provided the rheological component of that work. Oscillatory amplitude-sweep measurements were used to characterize the post-treatment rheological profile in terms of storage modulus, loss modulus, loss factor, complex viscosity, and the linear viscoelastic region [[1], [2], [3]]. Calcium-alginate hydrogels were selected because ionically crosslinked alginate networks provide a defined hydrated polymer system for assessing treatment-related rheological response [1,4,5]. The motivation for compiling this dataset was to make the complete point-by-point rheometer output available beyond the selected rheological plots and summarized parameters reported in the related article. This data article provides the raw measurement files and corresponding plots, allowing users to apply their own processing criteria, extract rheological parameters, and generate material profiles for untreated and treatment-exposed hydrogels.

## 3 Data Description

The Zenodo repository contains raw rheological measurement files and corresponding plots for untreated ionically crosslinked calcium-alginate hydrogels and hydrogels exposed to ultraviolet, ethylene oxide, steam, gamma, and high hydrostatic-pressure treatments [6]. The dataset comprises 13 experimental conditions and 39 individual amplitude-sweep datasets, with one sample from each of three independently prepared hydrogel batches measured per condition. The repository includes one README file and treatment-group archives. For each treatment group, the complete point-by-point rheometer export is available in CSV format, along with the corresponding rheological plots in PDF format. Gamma-treated samples are organized by dose, whereas high hydrostatic pressure-treated samples are organized by pressure level and number of cycles (Fig. 1).

**Fig. 1:**
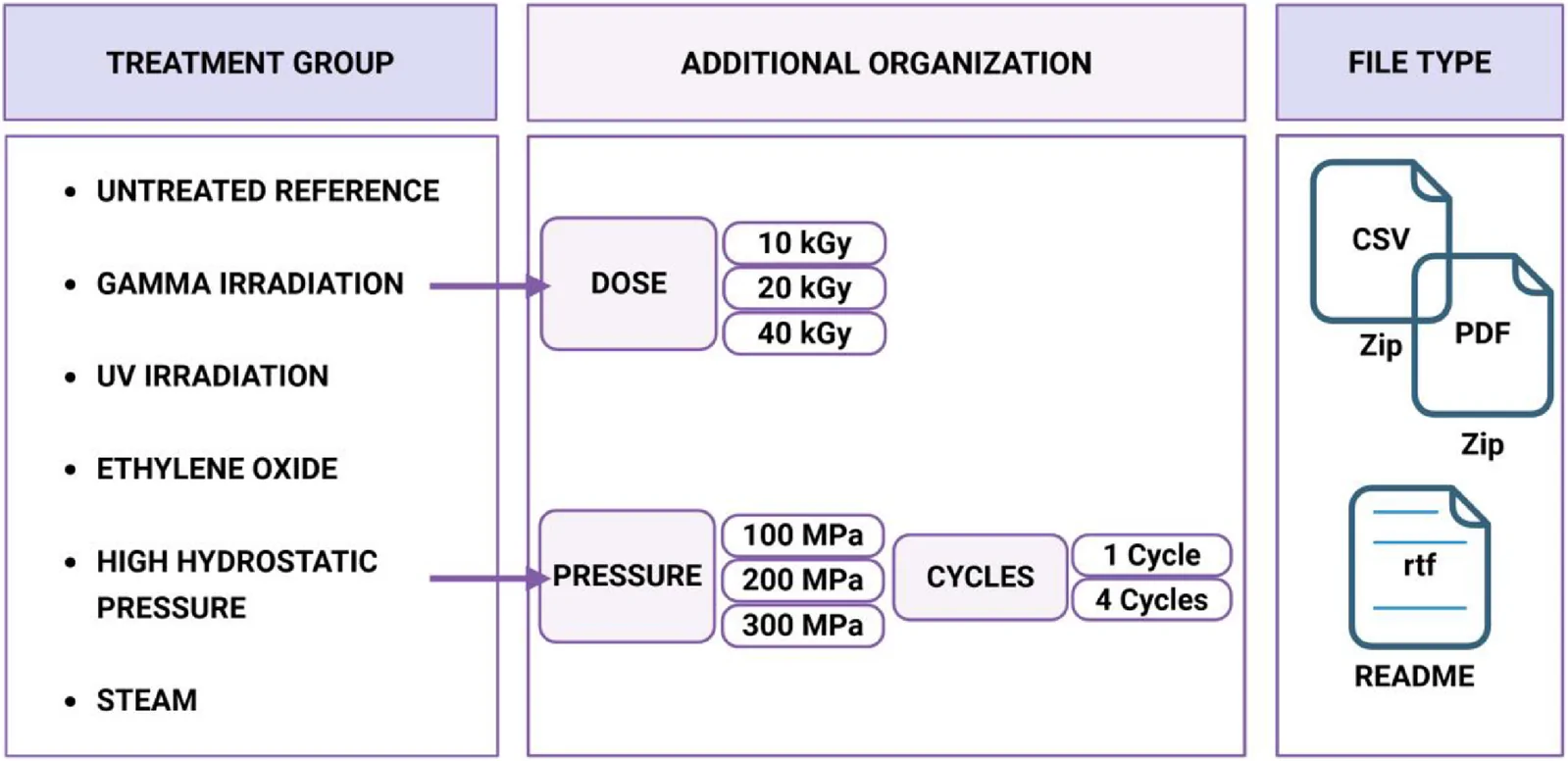
Organization of the rheological dataset in Zenodo.

Table 1 lists the treatment-specific raw data and corresponding figure archives included in the repository. The abbreviations used in the repository file names are UV, ultraviolet; EtO, ethylene oxide; and HHP, high hydrostatic pressure.

**Table 1:** Repository files and content of the Zenodo dataset.

Repository file	File type/format			Content
	RTF	ZIP/CSV	ZIP/PDF	
README_rheology_dataset_hydrogel.rtf	✓			Dataset title, keywords, description, folder structure, CSV variable definitions, method summary, reuse guidance, and contact information.
Unsterilized_csv.zip		✓		Raw amplitude sweep data for untreated calcium-alginate hydrogel reference samples.
Unsterilized_figures.zip			✓	Rheological plots for untreated reference samples.
UV_rheology_csv.zip		✓		Raw amplitude sweep data for ultraviolet-irradiated hydrogel samples.
UV_rheology_figures.zip			✓	Rheological plots for ultraviolet-irradiated hydrogel samples.
EtO_rheology_csv.zip		✓		Raw amplitude sweep data for ethylene oxide-treated hydrogel samples.
EtO_rheology_figures.zip			✓	Rheological plots for ethylene oxide-treated hydrogel samples.
Steam_sterilization_csv.zip		✓		Raw amplitude sweep data for steam-sterilized hydrogel samples.
Steam_sterilization_figures.zip			✓	Rheological plots for steam-sterilized hydrogel samples.
Gamma_sterilization_csv.zip		✓		Raw amplitude sweep data for gamma-irradiated hydrogel samples, organized by irradiation dose: 10, 20, and 40 kGy.
Gamma_sterilization_figures.zip			✓	Rheological plots for gamma-irradiated hydrogel samples, organized by irradiation dose.
HHP_rheology_csv.zip		✓		Raw amplitude sweep data for high hydrostatic pressure-treated hydrogel samples, organized by pressure level and cycle number.
HHP_rheology_figures.zip			✓	Rheological plots for high hydrostatic pressure-treated hydrogel samples, organized by pressure level and cycle number.

Each raw CSV file contains point-by-point amplitude sweep data, with each row corresponding to a measurement point recorded during the oscillatory rheology test. The exported variables and units are listed in Table 2. The deposited files contain the complete point-by-point CSV export, not a selected subset of variables.

**Table 2:** Variables included in raw rheology CSV files.

Column title in CSV file	Unit / format	Description
Point No.	Number	Measurement point index exported by the rheometer software.
Shear Strain	Dimensionless	Applied oscillatory shear strain recorded as a dimensionless ratio. Multiply by 100 to express the values as percentages, as shown in the corresponding PDF plots.
Shear Stress	Pa	Shear stress recorded at each strain point.
Storage Modulus	Pa	Elastic component of the oscillatory response, G′.
Loss Modulus	Pa	Viscous component of the oscillatory response, G″.
Loss Factor	Dimensionless	Ratio of loss modulus to storage modulus, tan δ = G″/G′, calculated by the rheometer software.
Torque	µN·m	Torque recorded by the rheometer during the measurement.
Complex Viscosity	mPa·s	Complex viscosity calculated by the rheometer software.
Status	Text	Instrument-generated status information retained in the rheometer export.

The corresponding PDF files provide graphical output generated from the same measurements. The plots show storage and loss moduli as functions of shear strain, storage and loss moduli as functions of shear stress, and complex viscosity as a function of shear strain. Fig. 2 shows representative rheological plots for one untreated calcium-alginate hydrogel sample and illustrates the plot types available in the repository.

**Fig. 2:**
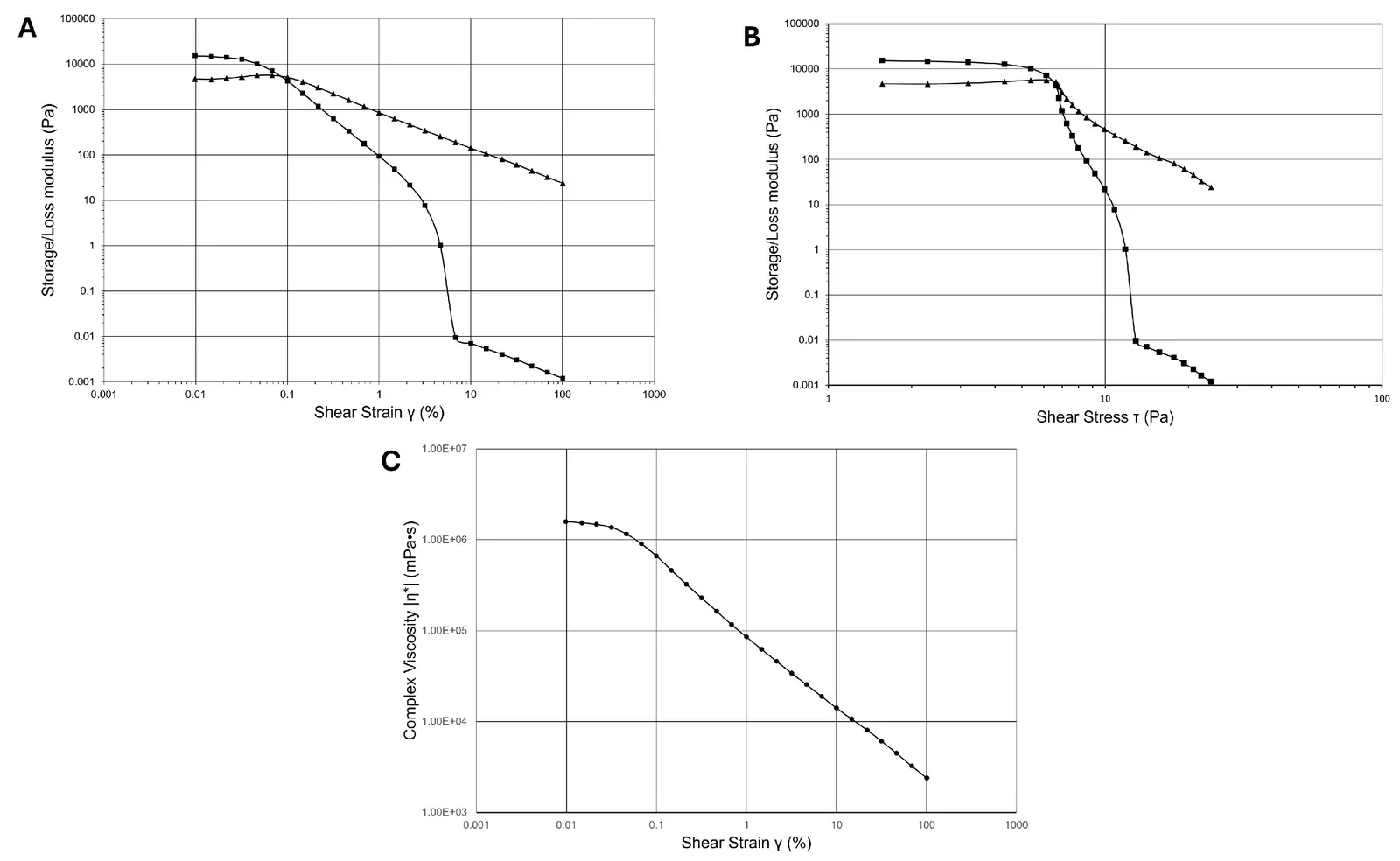
Representative rheological plots for an untreated calcium-alginate hydrogel, illustrating the types of graphical output provided with the dataset: (A) storage and loss moduli versus shear strain; (B) storage and loss moduli versus shear stress; and (C) complex viscosity versus shear strain. Corresponding instrument-generated plots are available as PDF files in the repository [6].

## 4 Experimental Design, Materials and Methods

Fig. 3 summarizes the workflow used to generate the rheological dataset. Pre-formed calcium-alginate hydrogel sections were used as untreated reference samples or exposed to ultraviolet, ethylene oxide, steam, gamma, or high hydrostatic pressure treatments. After treatment, all samples were analyzed by oscillatory amplitude sweep rheometry under identical measurement conditions. The rheometer output was exported for dataset preparation.

**Fig. 3:**
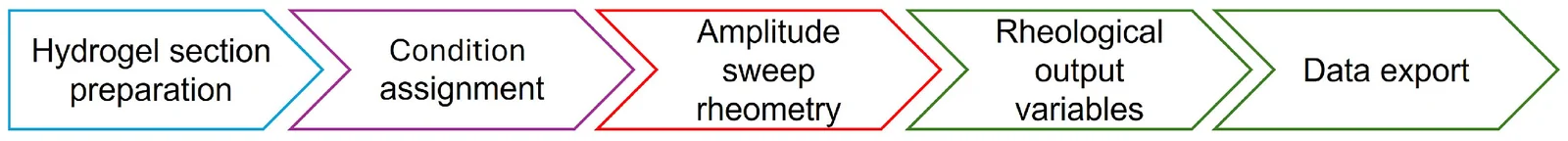
Workflow for generating the rheological dataset.

### 4.1 Hydrogel samples

Cylindrical calcium-alginate hydrogels were prepared as described in the related research article [1], using a diffusion-through-dialysis-tube approach adapted from Bajpai et al. [7]. Hydrogels were prepared from a 2% (w/v) sodium alginate solution, crosslinked by diffusion in a 1.5% (w/v) calcium chloride solution. Before loading into the dialysis tube, the sodium alginate solution was degassed to remove entrapped air bubbles. For each batch, 50 mL of alginate solution in dialysis tube (Membra-Cel™, MWCO 14,000) (from Dominique DUTSCHER SAS, Bernolsheim, France) was immersed in 450 mL of calcium chloride solution for 24 h at 22 ± 1 °C. The 24 h diffusion-crosslinking time was established in preliminary experiments and was kept constant for all batches. After crosslinking, the hydrogels were washed with distilled water. The same preparation conditions were used for all batches. Preliminary experiments were performed on 1 cm-thick sections cut from the hydrogel cylinders to assess preparation repeatability. Only hydrogel cylinders without visible defects on visual inspection were used for sectioning. The resulting cylinders were cut into 1 cm-thick sections before treatment and rheological measurement. Fig. 4 shows the hydrogel preparation configuration and representative sample geometry.

**Fig. 4:**
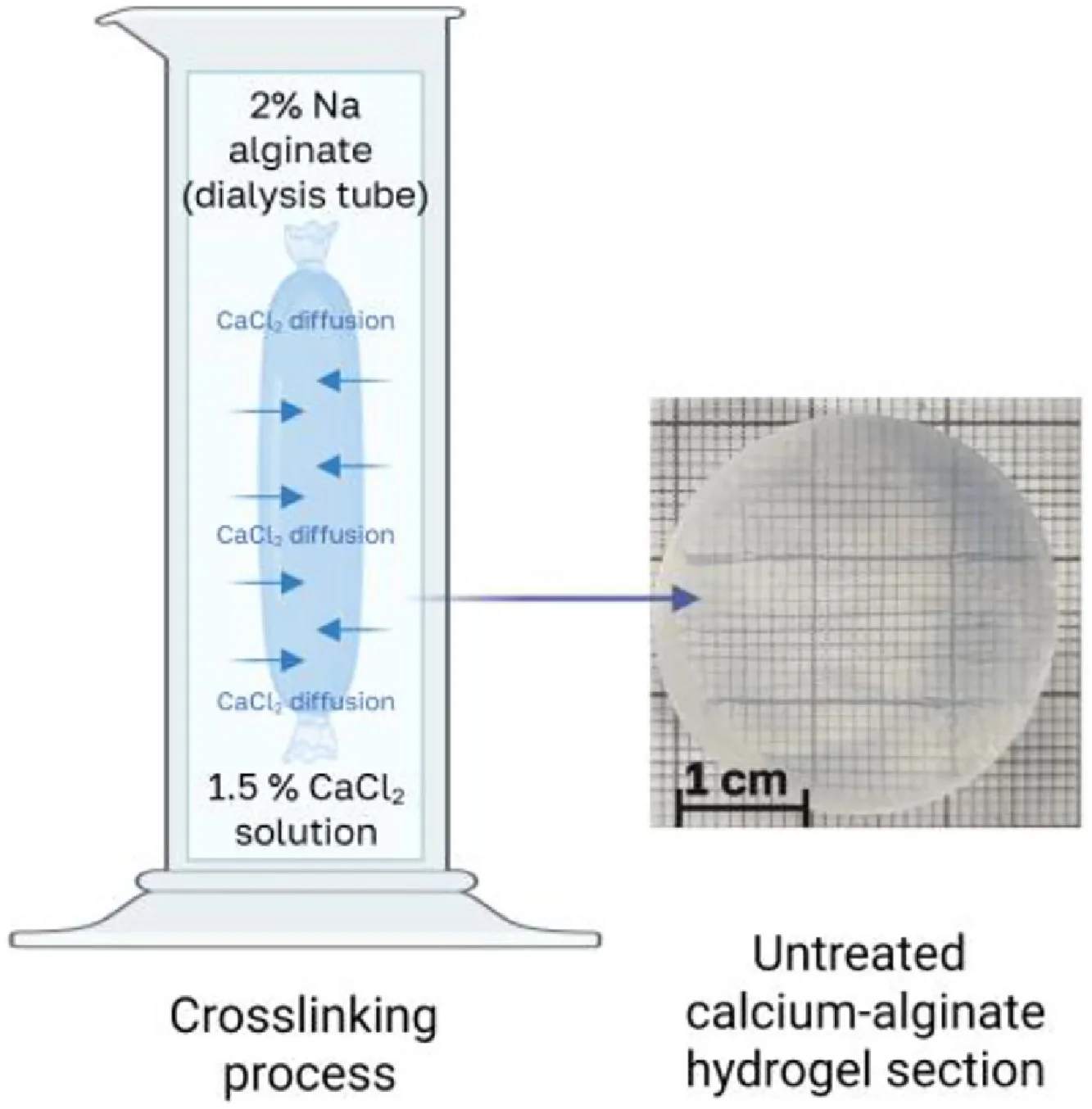
Calcium-alginate hydrogel preparation and sample geometry. Created in BioRender.

### 4.2 Treatment conditions

The sample set included untreated reference hydrogel sections and hydrogel sections processed using ultraviolet radiation, ethylene oxide, steam, gamma radiation, or high hydrostatic pressure. Reference sections were analyzed without sterilization or pressure processing. Ultraviolet exposure was carried out at 254 nm for 30 min per side at a distance of 20 cm using 1 cm-thick hydrogel sections. Ethylene oxide sterilization was conducted by a certified external provider using a validated industrial cycle in accordance with ISO 11,135, including post-treatment evacuation. Samples were returned within 3–5 days and measured within 24–48 h of receipt. Steam sterilization was conducted at 121 °C for 20 min under 179.5 kPa. Gamma-irradiated samples were processed by a certified external provider at nominal doses of 10, 20, and 40 kGy [1]. High hydrostatic pressure processing was performed at 100, 200, and 300 MPa, applied for one or four cycles, with a 10 min holding time per cycle [1,8].

### 4.3 Rheological measurements and data export

Rheological measurements were performed using an Anton Paar MCR 102e rheometer equipped with smooth 50 mm parallel plates and a Julabo Corio CP-200F temperature control system. Each hydrogel section was centered between the parallel plates, and the measurement gap was adjusted to 5 mm before testing. No normal-force control or additional gap correction was applied. The sections had a nominal thickness of 1 cm before treatment and were not cut to 5 mm before rheological measurement. Because treatment altered sample dimensions, adjustment to the fixed 5 mm gap resulted in treatment-dependent compression. Measurements were conducted at 37 °C. Samples were allowed to equilibrate at 37 °C for 10 min before measurement. Oscillatory amplitude sweep tests were performed on the same sample from low to high strain at a constant angular frequency of 10 rad s^−1^ over a shear strain range of 0.01–100%. Frequency-sweep measurements were not systematically acquired across the complete experimental design and are therefore not included in the dataset.

### 4.4 Data extraction and export

The rheometer software exported point-by-point values for shear strain, shear stress, storage modulus, loss modulus, loss factor, torque, and complex viscosity. Loss factor and complex viscosity were calculated by the rheometer software. Instrument-generated Status information was retained in the exported CSV files. The linear viscoelastic region was evaluated for the analysis reported in the related research article [1]; LVER was defined manually as the strain range over which G′ remained within ±5% of its low-strain plateau value. The low-strain plateau was identified from the initial stable region of the G′ curve. G″ was not used to define the endpoint. The same criterion was applied to all treatment conditions, and isolated fluctuations were not treated as the endpoint unless a sustained deviation was observed. However, the deposited CSV files contain the complete amplitude sweep output, allowing users to apply their own LVER criteria. Raw output files and the corresponding plots were exported separately for each sample. No datasets or individual measurement points were excluded during post-processing.

### 4.5 Measurement replicates

For each treatment condition, one 1 cm-thick hydrogel section from each of three independently prepared hydrogel batches was measured separately (n = 3). These samples therefore represent independent material-preparation replicates rather than technical replicates. For untreated controls, the between-batch RSD values were 14.7% for G′, 17.6% for G″, and 48.4% for LVER [1]. The related research article [1] reports statistical comparisons of derived rheological parameters, which are not repeated in this Data Article. The deposited dataset does not include preliminary repeatability measurements; it contains three untreated reference datasets.

## Limitations

The dataset is limited to rheological measurements of one calcium-alginate hydrogel formulation and one sample geometry. Accordingly, the results should not be generalized directly to other hydrogel formulations, crosslinking systems, or rheological measurement geometries. Each condition is represented by three independently prepared hydrogel batches (n = 3), which limits how precisely between-batch variability can be estimated.

The LVER showed greater variability than G′ and G″ in untreated controls [1]. The linear viscoelastic region is derived from amplitude sweep data and may vary depending on the selected data-processing criteria.

All complete amplitude-sweep datasets were acquired at a single angular frequency of 10 rad s^−1^; therefore, frequency-dependent viscoelastic behavior is not characterized.

No dedicated wall-slip or homogeneous-deformation tests were performed, and these effects cannot be completely excluded. Treatment-related changes in sample dimensions also resulted in different degrees of compression when samples were adjusted to the fixed 5 mm measurement gap.

Because the hydrogels were formed by diffusion-based Ca²⁺ crosslinking, spatial variations in Ca²⁺ concentration and crosslink density may have been present but were not mapped directly. To reduce preparation-related heterogeneity, the alginate solution was degassed before crosslinking, the diffusion time was fixed at 24 h, and only cylinders without visible defects were used. However, these measures do not demonstrate spatially uniform crosslinking. The 24 h diffusion time was established for the dialysis-tube geometry and crosslinking temperature used in this study. When reproducing the procedure with a different dialysis-tube diameter or crosslinking temperature, the diffusion time should be verified and adjusted accordingly.

UV exposure was standardized on both sides of the sections but did not establish uniform irradiation throughout the sample thickness.

For ethylene oxide- and gamma-processed samples, external processing, transport, and storage before rheological measurement may contribute to sample-level variability; for the ethylene oxide-treated samples, residual EtO was not quantified.

Rheological values were not normalized for water content or swelling. The rheological data alone cannot distinguish between polymer-chain scission, changes in crosslink density, ionic-network rearrangement, changes in water content, or other molecular mechanisms. Reversibility after rehydration or storage was not assessed.

## Ethics Statement

The authors confirm that they have read and comply with the ethical requirements for publication in Data in Brief. This work does not involve human subjects, animal experiments, or any data collected from social media platforms.

## Credit Author Statement

**Agnese Brangule:** Supervision, Conceptualization, Methodology, Writing – original draft preparation, Writing - review and editing, Data curation; **Paula Kaufelde:** Conceptualization, Methodology, Writing – review and editing, Investigation, Formal analysis, Data curation**; Anete Vircava:** Conceptualization, Methodology, Investigation, Formal analysis; **Konstantīns Logviss:** Writing – review and editing, Supervision, Resources.

## Declaration of Generative AI and AI-Assisted Technologies in the Manuscript Preparation Process

During the preparation of this work, the authors used Grammarly and ChatGPT 5.5 to refine the language and grammar. After using these tools, the authors reviewed and edited the content as needed and take full responsibility for the content of the published article.
